# Breastfeeding Practices Influence the Breast Milk Microbiota Depending on Pre-Gestational Maternal BMI and Weight Gain over Pregnancy

**DOI:** 10.3390/nu13051518

**Published:** 2021-04-30

**Authors:** Erika Cortés-Macías, Marta Selma-Royo, Cecilia Martínez-Costa, Maria Carmen Collado

**Affiliations:** 1 Department of Biotechnology, Institute of Agrochemistry and Food Technology, Spanish National Research Council (IATA-CSIC), 46980 Valencia, Spain; ercorma@iata.csic.es (E.C.-M.); mselma@iata.csic.es (M.S.-R.); 2 Department of Pediatrics, INCLIVA Research Institute, School of Medicine, University of Valencia, 46003 Valencia, Spain; cecilia.martinez@uv.es; 3 Pediatric Gastroenterology and Nutrition Section, Hospital Clínico Universitario Valencia, INCLIVA, 46010 Valencia, Spain

**Keywords:** body mass index, breastfeeding, microbiota

## Abstract

Breastfeeding is critical for adequate neonatal microbial and immune system development affecting neonate health outcomes in the short and long term. There is a great interest in ascertaining which are the maternal factors contributing to the milk microbiota and the potential relevance for the developing infant. Thus, our study aimed to characterize the effect of mixed and exclusive breastfeeding practices on the milk microbiota and to determine the impact of pre-pregnancy body mass index (BMI) and weight gain over pregnancy on its composition. Breast milk samples from 136 healthy women were collected within the first month post-partum and milk microbiota profiling was analyzed by 16S rRNA gene sequencing. Information on breastfeeding habits and maternal-infant clinical data were recorded. Breastfeeding practices (exclusive vs. mixed), maternal pre-gestational BMI, and weight gain over pregnancy contributed to the milk microbiota variation. Pre-gestational normal-weight women with exclusive breastfeeding habits harbored a significantly higher abundance of *Bifidobacterium* genus, and also, higher alpha-diversity compared to the rest of the women. Our results confirm the importance of controlling weight during pregnancy and breastfeeding practices in terms of milk microbiota. Further studies to clarify the potential impact of these maternal factors on milk and infant development and health will be necessary.

## 1. Introduction

Breast milk is the best and the primary food for infants since it covers the infant’s needs during a critical period of human development and health programming. Beyond nutritional aspects, it contains a diverse array of biologically active components which guide the adequate infant’s growth and development [1]. The World Health Organization [2] recommends exclusive breastfeeding during the child’s first six months to achieve optimal development and growth. Apart from several nutrients, human milk provides numerous types of bioactive compounds which support infant growth [3] and drives the development of the neonatal microbiota [4] and intestinal maturation [5].

Growing evidence suggests that breast milk microbiota would be influenced by different perinatal factors such as delivery mode, antibiotic treatment, diet, and maternal psychosocial status [6,7,8,9]. However, other studies did not report the effects of some of these variables on human milk [10,11,12]. Indeed, recent evidence has shown breastfeeding practices and milk collection methods would influence breast milk microbiota composition [9]. Results regarding the potential impact of maternal body mass index (BMI) and weight gain over pregnancy on breast milk microbiota are also still contradictory [13,14,15,16]. Similarly, while other studies found differences in milk microbiota according to lactation period [13,17] and feeding method [9], others reported a stable composition over time [18]. Therefore, it would be essential to provide evidence on the possible factors that shape the composition of breast milk microbiota. Thus, our study aimed to characterize the impact of the breastfeeding practices together with maternal age, pre-pregnancy BMI, and weight gain over pregnancy on the breast milk microbiota in a cross-sectional study.

## 2. Materials and Methods

### 2.1. Study Design and Mother-Infant Pairs

Healthy mother-infant pairs (*n* = 136) were included in the study (Supplementary Material, Figure S1). The participants gave their consent after receiving information, both oral and written. The participants were enrolled during the MAMI (Maternal Microbes, Valencia, Spain) birth cohort [19]. The protocol was approved by the Hospital Ethics Committees (Hospital Clínico Universitario de Valencia, Valencia, Spain). The study was also registered on the ClinicalTrial.gov platform (NCT03552939).

Clinical and anthropometric parameters including gestational age, maternal age, delivery mode, intrapartum antibiotic exposition, pre-gestational BMI, weight gain over pregnancy, and breastfeeding practices (exclusive breastfeeding, EBF, or mixed feeding, MF, a combination of breast milk and other milk) were collected. Regarding maternal age, participants were categorized into three age groups as following: <30 years, 30–35 years, and >35 years, corresponding approximately to the quartiles (Q1 = 32 and Q3 = 37 years).

Mothers were classified according to their pre-gestational BMI [20] as normal weight (NW) and overweight (OW) according to the WHO criteria [20]: NW (18.5–24.9 kg/m^2^) and OW (≥25.0 kg/m^2^). Additionally, mothers were also classified according to their weight gain over pregnancy according to their pre-gestational BMI following the recommendations of the Institute of Medicine [21] in normal weight gain (NWG) and excessive weight gain (EWG). Briefly, a NWG was considered as follows: 11.5–16.0 kg for NW mothers and 7.0–11.5 kg for OW mothers. Weight gain above the upper values of the recommended ranges was considered as EWG.

### 2.2. Breast Milk Samples and DNA Extraction

Breast milk samples were compiled within 30 days post-partum (*n* = 136). Breast milk collection was performed following a standardized protocol. Briefly, milk was collected using a sterile pumper in sterile bottles upon the cleaning of breast skin with 0.5% chlorhexidine solution. Milk samples were kept at −20 °C until its transport to the laboratory, where they were stored at −80 °C until further analysis.

Total DNA was extracted from 1.5–2.0 mL breast milk using the Master-Pure DNA Extraction Kit (Epicentre, Madison, WI, USA) as previously described [22]. DNA purification was performed by MagSi-NGS Plus kit (amsbio, Abingdon, UK) and was quantified using the Qubit 2.0 Fluorometer (Life Technology, Carlsbad, CA, USA). Controls during DNA extraction and PCR amplification were also included and sequenced.

### 2.3. Total Bacterial Load by qPCR

Total bacterial load was determined by qPCR amplification as described elsewhere [22]. Primers 789R (5′-GCGTGGACTACCAGGGTATCT) and 515F (5′-GTGCCAGCMGCCGCGGTAA) were use at annealing temperature of 62 °C [23,24]. Each reaction mixture of 10 μL was composed of SYBR^®^ Green PCR Master Mix (Roche), 0.25 μL of each of the specific primers in a LightCycler^®^ 480 real-Time PCR System (Roche Technologies, Basel, Switzerland), at a concentration of 10 μM, and 1 μL of template DNA. All amplifications were performed in duplicates. Standard curves for the targeted bacterial-amplicon were generated using Ct values and the calculated gene copies numbers were determined based on the fragment amplification length.

### 2.4. S rRNA Amplicon Sequencing and Bioinformatics

Breast milk microbiota composition was assessed by the sequencing of the V3–V4 variable region of the 16S rRNA gene following Illumina protocols as described by García-Mantrana et al. [25] on a MiSeq-Illumina platform (FISABIO sequencing service, Valencia, Spain). Briefly, Nextera XT Index Kit (Illumina, CA, USA) was used for the multiplexing step and libraries were sequenced using a 2 × 300 pb paired-end run (MiSeq Reagent kit v3).

Trimmomatic software [26] was used to search and remove the residual adaptors, and DADA2 pipeline v.1.16 [27] was performed for quality filtering, sequence joining, and chimera removal. Taxonomic assignment was achieved using the Silva v132 database [28] including the species-level classification. Additional filters were performed included those samples with less than 1000 reads and those amplicon sequence variants (ASV) with a relative abundance of less than 0.01% and those present in less than three times in at least 20% of the samples. Besides this, the decontam package [29] in the RStudio environment was used to identify possible contaminants and those were removed from the final analysis (*n* = 48 ASV). Sequence data have been deposited in the National Center for Biotechnology Information (NCBI) under the project accession number BioProject ID PRJNA614975.

### 2.5. Statistical Analysis

The following software was used for analysis: Calypso online platform (V8.84) [30], SPSS V.27 [31] (IBM Corp. Released 2020; IBM SPSS Statistics for Windows, Version 27.0. Armonk, NY: IBM Corp); and Graphpad Prism v. 5.04 (GraphPad Software, San Diego, CA, USA, www.graphpad.com accessed on 1 April 2021). Redundancy analysis (RDA) was applied to study the statistical effect of breastfeeding practices on breast milk microbiota. The differences between the groups were visualized by the discriminant of principal components analysis (DAPC) at the amplicon sequence variant (ASV) level and the Adonis test was achieved based on the Bray-Curtis distance.

*T*-test and Mann-Whitney analysis were used depending on data normality assessed by Kolmogorov-Smirnov and Shapiro-Wilk test (Graphpad Prism V5.04). Spearman correlations between relative abundances of bacterial and maternal age were using RStudio [32]. Multivariable Poisson regression models adjusted by covariables were run in SPSS v.27 to assess differential abundance at the genus levels (dependent variable), variable that consists of count data, according to pre-gestational BMI, weight gain, and breastfeeding practices (independent variable). The covariables that were used to adjust each model are specified in the description of their results.

## 3. Results

### 3.1. Subjects and Clinical Data

Maternal-infant characteristics are shown in Table 1. The average maternal age was 34.44 years. The median maternal pre-gestational BMI and weight gain over pregnancy were 22.84 kg/m^2^ and 12.0 kg, respectively. In the study cohort, 62.5% of deliveries were vaginal and 81.6% of the mothers followed the EBF practices during the first month of life.

We found significant differences in the total breastfeeding duration being significantly (*p* < 0.001) shorter in women with MF at 1 month (9.41 months vs. 3.76 months of duration, EBF and MF, respectively). In our cohort, 81.6% followed the EBF practices during the first month of life.

### 3.2. Factors Affecting Breast Milk Microbiota

**Figure 1:**
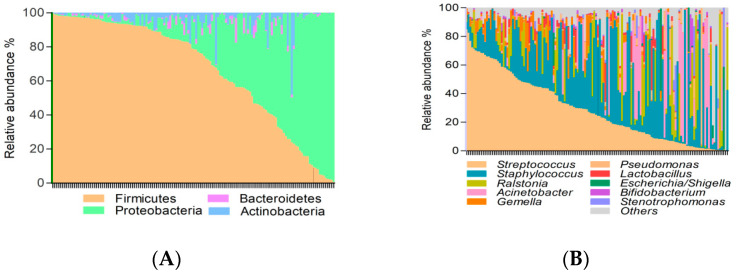

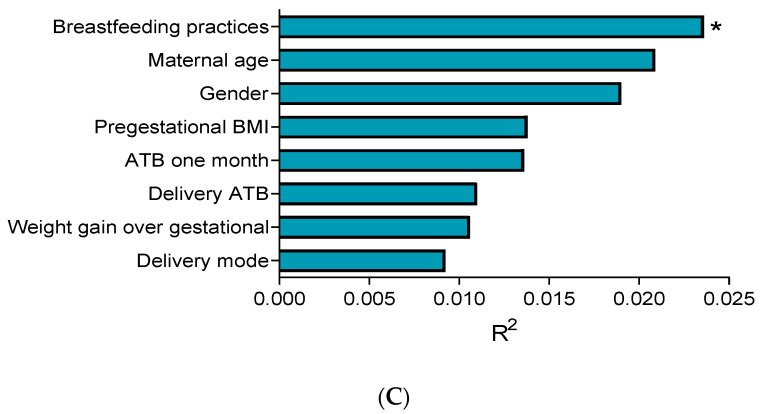
Breast milk microbiota composition 30-days after birth. Bar plot showing the relative abundances (%) at the phylum level (**A**) and of top 10 genera (**B**) detected in breast milk microbiota in each lactating mother. (**C**) Effect of perinatal factors on the breast milk microbiota composition assessed by Adonis test based on Bray-Curtis distance. * *p* < 0.05. Pre-gestational body mass index (BMI) and weight gain over pregnancy are numerical variables and the rest are categorical variables.

In general, the breast milk microbial communities were characterized by the dominance of Firmicutes (66.3%) and Proteobacteria (28.8%) phyla, followed by Actinobacteria (3.7%) and Bacteroidetes (1.18%) (Figure 1A). At the genus level, the taxa with higher relative abundance were *Streptococcus* (29.2%) and *Staphylococcus* (27.8%), followed by *Ralstonia* (10.1%) and *Acinetobacter* (9.6%) (Figure 1B).

A permutational multivariate analysis of variance Adonis, with Bray-Curtis distance metric, showed the main contributors to the breast milk microbiota variation (Figure 1C). Breastfeeding practices (*p* = 0.034) significantly affected the breast milk microbiota.

### 3.3. Breast Milk Microbiota Was Shaped by Breastfeeding Practices

**Figure 2:**
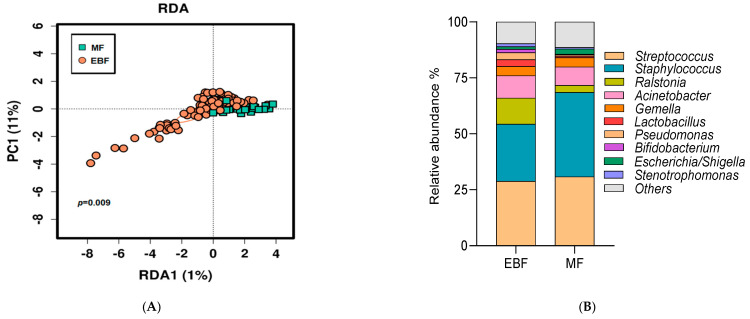

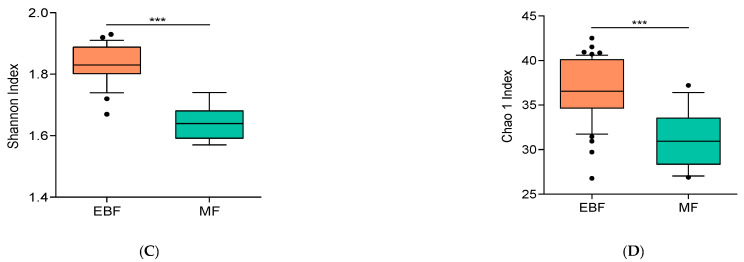
Impact of breastfeeding practices on breast milk microbiota. (**A**) Multivariate redundant discriminant analysis (RDA) at ASV level according to breastfeeding practices. (**B**) The relative abundance of bacteria present in breast milk at the genus level. (**C**,**D**) Microbial richness and diversity indexes at ASV. Richness and diversity values were adjusted for the total bacterial load. Whiskers represented 5–95 percentile intervals. *** *p* < 0.001. PC: principal components, EBF: Exclusive breastfeeding, MF: Mixed Feeding at one month.

Breast milk microbiota was significantly different according to breastfeeding practices (Adonis test *R*^2^ = 0.0174, and *p* < 0.01) at the ASV level. Indeed, breast milk microbiota was significantly different between those mothers that had or not an EBF in the multivariate analysis (RDA) (variance = 1.04, F = 1.73, *p* = 0.009, Figure 2A). Furthermore, distinct bacterial profiles at genus levels were observed in EBF and MF mothers (Figure 2B). At the genus level, EBF mothers a higher relative abundance of *Bifidobacterium* (*p* = 0.05), *Ralstonia* (*p* = 0.05) and *Pseudomonas* (*p* < 0.001) compared with MF mothers, was observed.

Regarding alpha-diversity, significantly higher bacterial richness (measured by Chao1 index) (*p* < 0.001) and diversity (measured by Shannon index) (*p* < 0.001) were observed in milk from EBF mothers compared to those observed in MF (Figure 2C,D).

In the Poisson regression models based on breastfeeding practices (EBF or MF) adjusted by covariates, including mode of delivery and antibiotic treatment for one month and pre-gestational BMI, breastfeeding practices were found to be consistently associated with some genera relative abundance (Table 2). EBF mothers had a higher prevalence of *Bifidobacterium* (9.52 (3.03–29.92)) and *Lactobacillus* (6.43 (3.42–12.10)) and lower incidence of *Staphylococcus* (0.69 (0.64–0.74)), and *Escherichia/Shigella* (0.42 (0.31–0.58)) in milk compared to MF mothers (Table 2). EBF mothers displayed a greater incidence of *Ralstonia* (incidence rate ratio (IRR): 3.72 (95% CI: 2.94–4.72)) in their milk compared to MF mothers (Table 2). Furthermore, the total bacterial counts in milk samples obtained by qPCR were not significantly different between the breastfeeding practices (EBF: 5.64 (4.77–6.17) vs. MF: 5.70 (4.94–6.34) Log10 bacterial gene copies/mL), *p* = 0.440).

### 3.4. Impact of Breastfeeding Practices on Breast Milk Microbiota Composition Was Modulated by Pre-Gestational BMI and Weight Gain over Pregnancy

**Figure 3:**
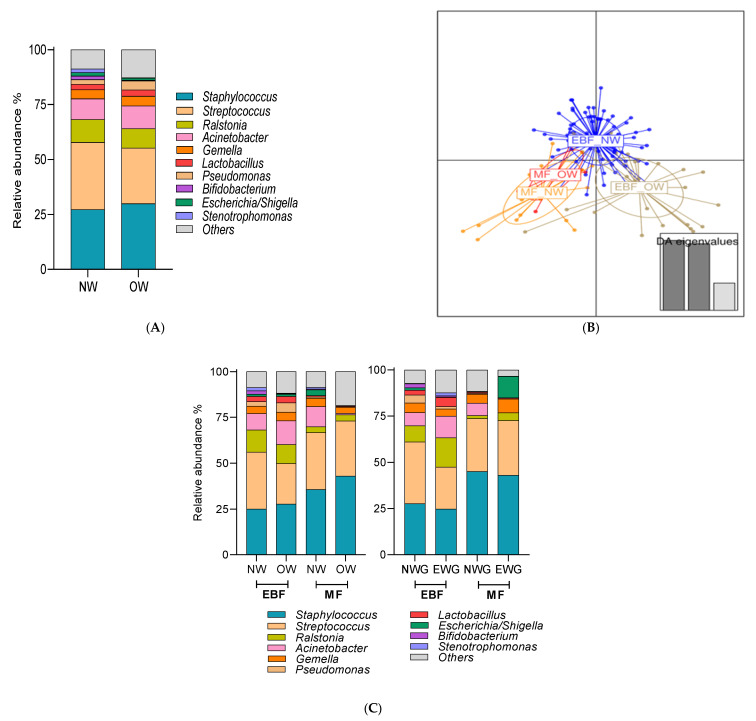
Breast milk microbiota was affected by breastfeeding practices depending on pre-gestational BMI and weight gain. (**A**) The relative abundance of bacteria present in breast milk at genus level according to pre-gestational BMI (**B**) Discriminant analysis of principal components (DAPC) of the breast milk microbiota according to the breastfeeding practices and pre-gestational BMI classification, and (**C**) the relative abundance of bacteria present in breast milk at genus level according to breastfeeding practices was modulated by pre-gestational and weight gain over pregnancy. Normal weight (NW), Overweight (OW). Excessive weight gain (EWG) and normal weight gain (NWG) according to pre-gestational BMI. Exclusive breastfeeding (EBF) and mixed feeding (MF) (breast milk and formula feeding).

We tested the impact of pre-gestational BMI and weight gain over pregnancy in the whole population. We observed that breast milk microbiota profile and also, alpha-diversity were influenced by pre-gestational BMI and weight gain. However, a higher relative abundance of *Bifidobacterium* (*p* = 0.029) genus was observed in NW mothers compared to OW mothers (Figure 3A). Regarding, the total bacterial counts in milk samples obtained by qPCR showed differences between NW mothers and OW mothers (NW: 6.70 (5.77–7.17) vs. OW: 6.94 (6.42–7.44) log10 bacterial gene copies/mL), *p* = 0.031).

In the Poisson regression models, maternal pre-gestational BMI was found to be consistently associated with the relative abundances of some specific genera NW mothers harbored a greater incidence of *Streptococcus* (incidence rate ratio (IRR): 1.21 (95% CI: 1.12–1.30)) in their milk compared to OW mothers (Supplementary Material, Table S1). However, NW mothers had a higher prevalence of *Bifidobacterium* (4.67 (2.53–8.64)) and *Ralstonia* (1.16 (1.03–1.32)) and a lower incidence of *Staphylococcus* (0.89 (0.83–0.96)) in milk compared to OW mothers (Supplementary Material, Table S1).

Due to the impact of breastfeeding practices on breast milk microbiota, we explored the possible potential effect of pre-gestational BMI and weight gain over the pregnancy in the milk microbiota (Supplementary Material, Table S2). Pre-gestational BMI and weight gain over pregnancy influenced the breast milk microbiota in a breastfeeding type-dependent manner. Pre-gestational BMI significantly impacted the breast milk microbiota composition in women with EBF practices (Adonis Bray–Curtis *R*^2^ = 0.0254, *p* = 0.05) while this observation was not reported in MF women (Adonis, Bray–Curtis *R*^2^ = 0.0537, *p* = 0.82). Furthermore, significant differences were observed in the whole microbial community between breastfeeding practices (EBF vs. MF) in NW mothers (RDA test variance = 1.47, *p* = 0.018; and Adonis, Bray–Curtis *R*^2^ = 0.022, *p* = 0.029), whereas no differences were detected in OW mothers (RDA test variance = 1.56, *p* = 0.928; Adonis, Bray–Curtis *R*^2^ = 0.0183, *p* = 0.78) (Supplementary Material, Figure S2). Discriminant analysis of principal components (DAPC) showed a distinct cluster of NW mothers with EBF compared to the other women (Figure 3B). Indeed, distinct bacterial profiles at the genus level by pre-gestational BMI and breastfeeding practices were observed (Figure 3C and Supplementary Material, Table S3). Higher relative abundance of *Bifidobacterium* (*p* = 0.033) genus were found in EBF_NW mothers compared to the other groups and significantly lower abundances of *Pseudomonas* (*p* < 0.01) was observed in MF_OW mothers compared to the other groups.

Regarding the effect of weight gain on breast milk microbiota, distinct bacterial profiles at the genus level according to weight gain over pregnancy and breastfeeding practices were observed (Figure 3C and Supplementary Material, Table S3). Significant differences were observed in the whole microbial community between breastfeeding practices only in NWG mothers (RDA test variance = 1.3, *p* = 0.014; and Adonis Bray–Curtis *R*^2^ = 0.015, *p* = 0.111), whereas no differences were detected in EWG mothers according to breastfeeding practices (RDA test variance = 1.43, *p* = 0.087; Adonis Bray–Curtis *R*^2^ = 0.0189, *p* = 0.109) (Supplementary Material, Figure S2). Higher *Staphylococcus* (*p* = 0.049) genus and lower relative abundances of *Pseudomonas* (*p* = 0.019) genus were found in MF_NWG mothers compared to the other groups (Figure 3C and Supplementary Material, Table S3).

In the Poisson regression modeling, NWG mothers displayed a greater incidence of *Streptococcus* in their milk compared to EWG mothers (incidence rate ratio (IRR): 1.38 (95% CI: 1.27–1.51)) (Supplementary Material, Table S1). Contrary, mothers classified as NWG showed a higher incidence of *Bifidobacterium* (3.20 (1.71–5.98)) and lower incidence of *Ralstonia* genus(0.53 (0.46–0.61)) in breast milk samples as the incidences observed in the EWG mothers which showed a higher relative abundance of *Escherichia/Shigella* genus (0.19 (0.14–0.26)) (Supplementary Material, Table S3). Further, the total bacterial counts displayed no statistically significant differences between NWG and EWG (NWG: 6.79 (5.80–7.25) vs. EWG: 6.69 (5.89–7.34) log10 bacterial gene copies/mL), *p* = 0.926).

**Figure 4:**
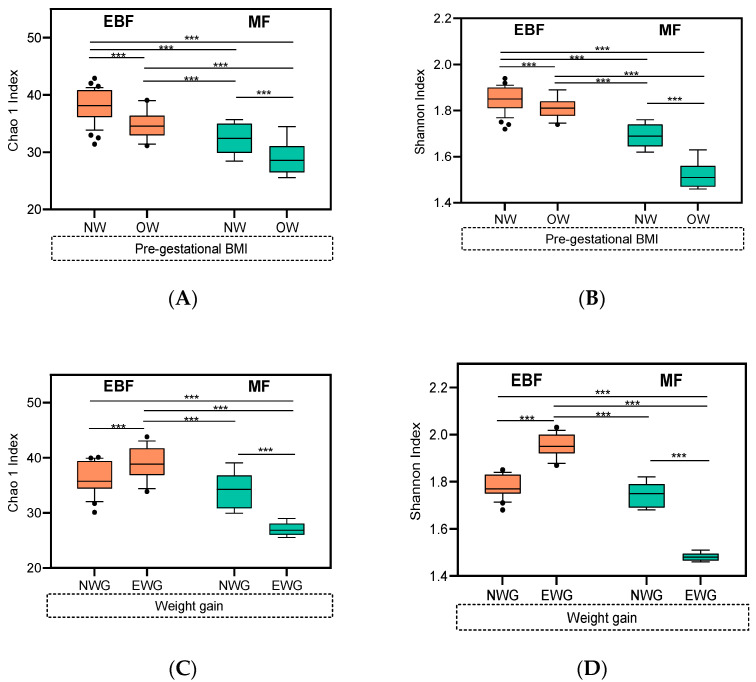
Alpha-diversity on the breast milk microbiota is associated with breastfeeding practices modulated by pre-gestational BMI and weight gain over pregnancy. Alpha diversity indexes: Diversity (Shannon index) and richness (Chao1 index) indexes at ASV of breast milk microbiota according to (**A**,**B**) pre-gestational BMI and (**C**,**D**) weight gain over pregnancy. Richness and diversity values were adjusted for the total bacterial load. Whiskers represented 5–95 percentile intervals. *** *p* < 0.001. NW: Normal weight, OW: Overweight, EWG: Excessive weight gain, NWG: Normal weight gain.

An association between the diversity and richness of breast milk microbiota and breastfeeding practices modulated by pre-gestational BMI and weight gain over pregnancy was found. Higher microbial diversity and richness were observed in women from EBF and NW mothers compared to the other groups (Figure 4A,B). Regarding the effect of these variables in microbial diversity and richness on weight gain over pregnancy breastfeeding practice and weight gain over pregnancy (Figure 4C,D), higher microbial diversity and richness were observed in women from MF and NWG mothers compared to the other groups.

A relationship between the diversity and richness of breast milk microbiota, pre-gestational BMI, and weight gain over the pregnancy is presented (Supplementary Material, Figure S3). Significant higher bacterial richness (*p* < 0.001) and diversity (*p* < 0.001) were observed in NW compared to OW mothers (Supplementary Material, Figure S3). Further, significantly lower bacterial diversity (*p* = 0.026) was observed in NWG mothers to EWG mothers, but this difference was not statistically significant in the case of bacterial richness (Supplementary Material, Figure S3). Besides this, lower microbial diversity measured by Shannon index was associated to higher pre-gestational (*ρ* = −0.05, *p* = 0.582) and lower weight gain over pregnancy (*ρ* = 0.05, *p* = 0.540), and also, lower microbial richness was associated to higher pre-gestational BMI (*ρ* = −0.03, *p* = 0.753) and weight gain over pregnancy (*ρ* = −0.01, *p* = 0.918).

## 4. Discussion

Breastfeeding practices, EBF vs. MF, have a key impact on the composition and diversity of the breast milk microbiota. Likewise, we found that pre-gestational BMI and weight gain over pregnancy are related to the composition and, especially, the diversity of breast milk microbiota. Our results confirm the importance of breastfeeding practices and also, maternal factors that would shape the milk microbial composition.

Breastfeeding, maternal diet, pre-gestational BMI, weight gain over pregnancy, maternal age, genetics, and geographical location have been reported to influence breast milk microbial composition [8,9,15,17]. We have previously reported the impact of maternal diet on breast milk microbiota [8] in the same population being the fiber, and that both plant and animal proteins are the most important contributors.

Nevertheless, the impact of these factors and others, such as breastfeeding type, pollution exposure, maternal age, parity, and others, on breast milk microbial communities is still unclear. EBF during the first months of life is important for the growth and development of the child [33]. Breastfeeding provides prebiotics such as human milk oligosaccharides, to support the developing infant gut microbiota [34]. As a component of breast milk, the microbiota associated with mothers’ milk contributes to the initial intestinal microbiota colonization of infants, having also a pivotal role in modulating the newborn’s immune system maturation [35]. Also, EBF contributes to protection against common infections during infancy [36]. Besides this, EBF was found to partially restore gut microbiota in cesarean-delivered infants [37]. We noted similar results between our findings concerning breastfeeding practices in terms of milk microbial composition and previous reports on the development of the gut microbiota and oral microbiota [38,39,40]. Among them, the increased relative abundance of *Bifidobacteriaceae* and *Enterobacteriaceae* in infants who were EBF [39], though, lower gut microbial diversity was observed in infants who were EBF [38,39]. Regarding infant oral microbiota it was reported to differ between EBF and formula-fed three-month-old infants, with Lactobacilli being cultured from exclusively and partially breastfed infants, but not from formula-fed infants [41]. It has been shown that breastfed neonates had higher microbial diversity than those infant fed with formula at four months of life and the impact is maintained during the first years of life [42,43,44], and also, different trajectories were found between breastfed and partially breastfed on oral microbiota up to seven years of life [43]. However other studies did not report differences in oral microbiota between breastfed and formula-fed infants [45].

The differences in breast milk microbiota composition and diversity according to breastfeeding practices would be partially explained by the impact of the infant oral microbiota which is influenced by mixed/exclusive breastfeeding practices as described previously [41,46]. Other potential factors would also include the impact of method of milk extraction (pumping vs. direct breastfeeding), and breastfeeding mode (direct vs. some indirect) on breast milk microbiota is relevant for microbial communities [9]. Data showed differences in composition towards an increase in the presence of potential pathogens on pumped milk compared to breast milk, as well as lower richness associated with indirect vs. direct breastfeeding. Moreover, shifts in maternal skin-areola microbiota due to less infant contact, and also, the potential maternal hormonal or physiological factors due to the reduction of exclusive feeds within a day, maternal stress among others would also influence the microbial communities in milk. We could not also exclude the impact of other environmental factors, such as pollution, siblings, and pets at home, that would influence both microbiota, mothers, and their infants, that would also contribute to the milk microbiota shifts. Indeed, our data showed that EBF practices at one month shaped breast milk microbiota composition and diversity. Higher microbial richness and diversity were observed in EBF [9,46]. Furthermore, as it was found in our study, mothers who fed their infants exclusively with human milk had an increased incidence of Acinetobacter in their milk [46]. Interestingly, we found a higher prevalence of Bifidobacterium and Lactobacillus genera in breast milk samples from mothers that followed an EBF compared to those following an MF. These genera are considered as typical components of gut microbiota from healthy, breastfed infants [35,47], and differences in their relative abundance could have important implications for infant immune development and the establishment of the newborn microbiota [47]. Regarding the possible health consequences of these observations, studies with infants that have had a long EBF showed better health outcomes, including the reduction of diarrhea-related gut microbiota dysbiosis as well as long-lasting gut microbiota differences compared to formula-fed infants [48].

Regarding maternal nutritional status, in our study, pre-gestational BMI was associated with the relative abundance of some genera as well as with microbial diversity and richness. Previous studies have reported associations between pre-gestational BMI and breast milk microbial composition [49]. In accordance with our results, breast milk from obese mothers has been reported to harbor a less diverse bacterial community compared to that found in NW mothers [13]. Other studies identified a higher incidence of members from Bacteroidetes phylum and lower incidence of members of Proteobacteria, a pro-inflammatory phylum, in breast milk at three months post-partum in obese mothers compared to those incidences observed in OW mothers [49]. Furthermore, a higher relative abundance of *Staphylococcus* genus has been observed in breast milk microbiota from women who exhibited an obese pre-pregnancy BMI [49]. In this sense, we found that the *Staphylococcus* genus was also associated with OW mothers while *Bifidobacterium* was associated with NW mothers compared to OW. Similarly, another study reported lower *Bifidobacterium* relative abundance on the gut microbiota of OW mothers even though they reported increased levels of *Staphylococcus*, and *Escherichia coli* in OW women compared to those NW women [50]. In our results, the qPCR median values of breast milk at 30 days were around 10^5^–10^6^ bacterial gene copies per mL in agreement with other studies performed using culture-independent techniques [22]. However, other studies based on traditional culture methodology reported lower bacterial densities of 10^3^–10^4^ per mL of breast milk [51]. These observations would be related to the potential presence of free DNA or inactive bacterial cells in milk. Indeed, our results showed significant differences in the total bacterial load according to pre-gestational BMI, corroborating the conception that weight changes shape the breast milk microbiota composition and diversity. In agreement with our results, Cabrera-Rubio et al. described differences in the proportion of specific taxonomic groups according to pre-gestational BMI [13]. Our findings clearly suggest that milk from NW mothers was associated with a higher relative abundance of *Streptococcus* and *Bifidobacterium* genera. Similarly, previous studies have been reported that OW mothers had lower levels of *Bifidobacterium* in their milk microbiota [52]. However, while some studies have reported results in agreement with our findings, others, observed no influence of pre-gestational BMI on the breast milk microbiota composition and diversity [9]. In this sense, Li et al. found no differences according to BMI in the most predominant bacterial families in the breast milk microbiota from East Asian women [53]. Besides this, our results also suggest that mothers who had an NWG mother displayed a greater incidence of *Streptococcus* and *Bifidobacterium* in their milk compared to EWG mothers. Colonization with the *Bifidobacterium* genus has been associated with several positive health outcomes in observational studies [54] and it has been highlighted as one of the most important genera for infant microbiota during the first months of life [55]. EWG has been related to an increased risk of high birth weight babies, large for gestational age, development of hypertension, preeclampsia, and emergency cesarean deliveries in both NW and OW women [56]. Despite its relationship with health outcomes, its possible effect on breast milk microbiota has been underexplored and the available data is still scarce. Besides this, our results showed that weight gain during pregnancy have an impact on the relative abundance of some genera as well as on microbial diversity. Breast milk samples from NWG mothers showed a lower diversity than those from EWG mothers.

Considering the impact of breastfeeding practices on milk microbiota depending on pre-gestational maternal BMI and weight gain over pregnancy, we found an increase in the abundance of *Bifidobacterium* genus in the breast milk of mothers from the EBF_NW group. Indeed, we found that mothers with EBF and NW were characterized by higher diversity and richness. These data are in agreement with previous studies [57]. Other studies reported that pre-gestational BMI maternal and gestational weight gain were each independently associated with the duration of breastfeeding [58]. These results suggested that the possible interaction between breastfeeding practices and pre-gestational BMI has an effect on breast milk microbiota.

In our study, maternal age was associated with the relative abundance of some genera human milk microbiota. We found a negative correlation between *Staphylococcus* genera and maternal age in mothers from the <30 group and 30–35 years group. However, a positive correlation between maternal age and *Streptococcus* genus was observed in older mothers (>35 years) (Supplementary Figure S4). A significant difference in diversity was observed based on the maternal age group, with older mothers (ages 30–35) having a higher Shannon diversity index (*p* < 0.001) in their breast milk microbiota compared to those from younger mothers (Supplementary Figure S4). However, these results need to be carefully considered since most of the volunteers in our study were in the range of 30–40 years (85%). Thus, the characteristics of the analyzed cohort, make this is not suitable for the assessment of the effect of maternal age on breast milk microbiota. Indeed, the impact of maternal age was lost when the mothers were grouped according to their age. However, our results based on the small number of mothers with the lowest and highest ages suggest that substantial physiological changes associated with aging could impact the breast milk microbiota. In accordance with our results, another study observed that maternal age influenced the diversity of breast milk bacterial profiles, showing that mothers with ages ≥35 years had a higher Shannon diversity index [59]. Nevertheless, another study showed that maternal age was not associated with bacterial breast milk [9], though, Padilha et al. demonstrated that manipulating the human milk microbiota through prebiotics is possible and that maternal age can affect this response [60]. Our results and others suggest a possible role of maternal age on breast milk microbiota and thus, further studies performed in cohorts with higher ranges of ages would be needed to clarify this effect. Thus, perinatal factors that affect its presence and abundance in breast milk microbiota could also indirectly impact the infant’s development through the breastfeeding process. Therefore, further research is needed to clarify the impact of these maternal factors on the breast milk microbiota.

This study has some limitations, including the limited sample size which reduces the possibility of detecting other significant associations as well as the lack of obese mothers. Our study is a part of an observational birth cohort and we identified that 27.9% of our population was overweight. In general, the maternal pre-gestational BMI was normal weight, thus further studies aimed to analyze the impact of breastfeeding practices and microbiota according to different BMI and weight gain are needed. Furthermore, we also evaluated these relationships in the first postpartum month period, and the results obtained need to be studied at other time points as well as during lactation. However, even if associations could be modified over lactation, these relations would be established in an extremely important period of infant maturation and therefore, they may impact the child’s development with long-lasting consequences. Moreover, most of the studies focused on breast milk microbiota have relied on small cohorts and thus their findings require replication in larger studies. Our study reported data from 136 women but longitudinal data, as well as a bigger cohort, are needed in parallel to the identification of the potential impact on infant development and health outcomes. Further, differences in sample handling protocols, collection procedures and processing data pipelines could also be the source of variability and act as confounding factors.

Despite all those potential limitations, our results shed light on the effect of some maternal factors on the composition and diversity of the breast milk microbiota, mainly the effect of breastfeeding practices, as limited evidence is available. Despite the importance of breastfeeding for infant growth, factors that modulate the microbial composition of breast milk have been underexplored. Therefore, efforts should be taken to ascertain the exact mechanisms by which maternal health and related factors could influence the breast milk microbiota to open the door to the design of strategies that target the modulation of breast milk microbiota composition and diversity with consequences in infant health, and in this way verify if the difference in the composition of the microbiota of breast milk has clinical relevance.

## Figures and Tables

**Table 1:** Characteristics of volunteers included in the analysis (*n* = 136).

	All	Exclusive Breastfeeding (*n* = 111)	Mixed Feeding (*n* = 25)	*p*
Maternal characteristics				
Maternal age (years)	34.44 ± 3.79	34.39 ± 3.84	34.68 ± 3.61	0.728
Gestational age (weeks)	40 (39,40)	40 (39–41)	39 (39,40)	0.005
Pre-gestational BMI (Kg/m^2^) *	22.84 (21.01–25.39)	22.62 (20.96–25.42)	23.01 (21.29–25.60)	0.386
Normal weight (NW)	97 (71.3)	79 (71.2)	18 (72.0)	0.985
Over weight (OW)	38 (27.9)	31 (27.9)	7 (28.0)	
Weight gain (kg) over pregnancy ^ǂ^	12 (9.5–15)	12 (10–15)	10 (7.8–14)	0.874
Low weight gain (LWG)	43 (31.6)	35 (31.5)	8 (32.0)	0.876
Normal weight gain (NWG)	60 (44.1)	48 (43.2)	12 (48.0)	
Excessive weight gain (EWG)	32 (23.5)	27 (24.3)	5 (20.0)	
Intrapartum antibiotic exposure (%)	54 (39.7)	46 (41.4)	8 (32.0)	0.383
Antibiotic during pregnancy (%)	42 (30.9)	34 (30.6)	8 (32.0)	0.915
Antibiotic treatment during 1 month (%)	11 (8.1)	8 (7.2)	3 (12.0)	0.435
Infant characteristics				
Gender: Female (%)	76 (55.9)	59 (53.2)	17 (68.0)	0.177
Birth mode: vaginal birth (%)	85 (62.5)	68 (61.3)	17 (68.0)	0.530
Height at birth (cm)	49.86 ± 2.10	50.12 ± 2.07	48.74 ± 1.89	0.003
Weight at birth (g)	3.32 ± 0.44	3.36 ± 0.42	3.12 ± 0.49	0.013
Antibiotic treatment 1 month (%)	8 (5.9)	7 (6.3)	1 (4.0)	0.658
Total breastfeeding duration (months)	8.37 ± 3.92	9.41 ± 3.24	3.76± 3.32	<0.001

Categorical data results are shown as the number of cases (percentage %). Normally distributed data are shown as mean ± SD and non-normal data as median (interquartile range = IQR). Normal weight (NW), overweight (OW), according to their pre-gestational BMI [21]. Normal weight gain (NWG), excessive weight gain (EWG) according to the categories from the Institute of Medicine [22]. * One mother without BMI information (*n* = 1) was not included. ^ǂ^ Low weight gain (LWG) data were not used in the analysis.

**Table 2:** Poisson regression model for top 10 genera relative abundance based on breastfeeding practices one month.

		Breastfeeding Practices One Month			
Genus	Rel.abund (%)		IRR	95% CI	*p*
*Streptococcus*	29.19	EBF vs. MF	0.92	0.85–1.00	0.033
*Staphylococcus*	27.78	EBF vs. MF	0.69	0.64–0.74	<0.001
*Ralstonia*	10.11	EBF vs. MF	3.72	2.94–4.72	<0.001
*Acinetobacter*	9.64	EBF vs. MF	1.26	1.08–1.47	0.003
*Gemella*	4.20	EBF vs. MF	0.95	0.76–1.20	0.678
*Pseudomonas*	2.69	EBF vs. MF	9.60	4.53–20.28	<0.001
*Lactobacillus*	2.57	EBF vs. MF	6.43	3.42-12.10	<0.001
*Escherichia/Shigella*	1.40	EBF vs. MF	0.42	0.31–0.58	<0.001
*Bifidobacterium*	1.25	EBF vs. MF	9.52	3.03–29.92	<0.001
*Stenotrophomonas*	1.23	EBF vs. MF	1.33	0.77–2.28	0.303

Poisson regression models were run for the top 10 genera based on breastfeeding practices, adjusted for the mode of birth and antibiotic treatment at one month and pre-gestational BMI. *p* < 0.05 was considered statistically significant. Incidence rate ratio (IRR). Confidence interval (CI). Exclusive breastfeeding (EBF) and mixed feeding (MF) at one month.

## Data Availability

Sequence data have been deposited in the National Center for Biotechnology Information (NCBI) under the project accession number BioProject ID PRJNA614975.

## Supplementary Materials

The following are available online at https://www.mdpi.com/article/10.3390/nu13051518/s1, Figure S1: Flow diagram of study participants. * Low weight gain (LWG) data were not used in the analysis, Figure S2: Impact of pre-gestational BMI and weight gain over pregnancy on breast milk microbiota according to breastfeeding practices, Figure S3: Microbial richness and diversity is associated by pre-gestational BMI and weight gain influence. Diversity (Shannon index) and richness (Chao1 index) of breast milk microbiota according to pre-gestational BMI and weight gain categories, Figure S4: Impact of maternal age on breast milk microbiota, Table S1: Poisson regression model for top 10 genera relative abundance based on pre-gestational BMI and weight gain over pregnancy, Table S2: Characteristics of population participant based pre-gestational BMI (*n* = 135), Table S3: Comparison of breast milk microbiota composition at top 10 genera, by breastfeeding practices and pre-gestational BMI and Weight gain.

## References

[1] Le Doare K., Holder B., Bassett A., Pannaraj P.S. (2018). Mother’s Milk: A purposeful contribution to the development of the infant microbiota and immunity. Front. Immunol..

[2] World Health Organization (2011). Exclusive Breastfeeding for Six Months Best for Babies Everywhere.

[3] Hoppu U., Kalliomäki M., Laiho K., Isolauri E. (2001). Breast milk—Immunomodulatory signals against allergic diseases. Allergy Eur. J. Allergy Clin. Immunol..

[4] Pastor-Villaescusa B., Hurtado J.A., Gil-Campos M., Uberos J., Maldonado-Lobón J.A., Díaz-Ropero M.P., Bañuelos O., Fonollá J., Olivares M., the PROLAC Group (2020). Effects of Lactobacillus fermentum CECT5716 Lc40 on infant growth and health: A randomised clinical trial in nursing women. Benef. Microbes.

[5] Jakaitis B.M., Denning P.W. (2014). Human breast milk and the gastrointestinal innate immune system. Clin. Perinatol..

[6] Hermansson H., Kumar H., Collado M.C., Salminen S., Isolauri E., Rautava S. (2019). Breast milk microbiota is shaped by mode of delivery and intrapartum antibiotic exposure. Front. Nutr..

[7] Browne P.D., Aparicio M., Alba C., Hechler C., Beijers R., Rodríguez J.M., Fernández L., de Weerth C. (2019). Human milk microbiome and maternal postnatal psychosocial distress. Front. Microbiol..

[8] Cortes-Macías E., Selma-Royo M., García-Mantrana I., Calatayud M., González S., Martínez-Costa C., Collado M.C. (2021). Maternal diet shapes the breast milk microbiota composition and diversity: Impact of mode of delivery and antibiotic exposure. J. Nutr..

[9] Moossavi S., Sepehri S., Robertson B., Bode L., Goruk S., Field C.J., Lix L.M., de Souza R.J., Becker A.B., Mandhane P.J. (2019). Composition and variation of the human milk microbiota are influenced by maternal and early-life factors. Cell Host Microbe.

[10] Williams J.E., Carrothers J.M., Lackey K.A., Beatty N.F., York M.A., Brooker S.L., Shafii B., Price W.J., Settles M.L., McGuire M.A. (2017). Human milk microbial community structure is relatively stable and related to variations in macronutrient and micronutrient intakes in healthy lactating women. J. Nutr..

[11] Drago L., Toscano M., De Grandi R., Grossi E., Padovani E.M., Peroni D.G. (2017). Microbiota network and mathematic microbe mutualism in colostrum and mature milk collected in two different geographic areas: Italy versus Burundi. ISME J..

[12] Urbaniak C., Angelini M., Gloor G.B., Reid G. (2016). Human milk microbiota profiles in relation to birthing method, gestation and infant gender. Microbiome.

[13] Cabrera-Rubio R., Collado M.C., Laitinen K., Salminen S., Isolauri E., Mira A. (2012). The human milk microbiome changes over lactation and is shaped by maternal weight and mode of delivery. Am. J. Clin. Nutr..

[14] Lundgren S.N., Madan J.C., Karagas M.R., Morrison H.G., Hoen A.G., Christensen B.C. (2019). Microbial communities in human milk relate to measures of maternal weight. Front. Microbiol..

[15] Gomez-Gallego C., Garcia-Mantrana I., Salminen S., Collado M.C. (2016). The human milk microbiome and factors influencing its composition and activity. Semin. Fetal Neonatal Med..

[16] Butts C.A., Paturi G., Blatchford P., Bentley-Hewitt K.L., Hedderley D.I., Martell S., Dinnan H., Eady S.L., Wallace A.J., Glyn-Jones S. (2020). Microbiota composition of breast milk from women of different ethnicity from the Manawatu—Wanganui region of New Zealand. Nutrients.

[17] Wan Y., Jiang J., Lu M., Tong W., Zhou R., Li J., Yuan J., Wang F., Li D. (2020). Human milk microbiota development during lactation and its relation to maternal geographic location and gestational hypertensive status. Gut Microbes.

[18] Shenker N.S., Perdones-Montero A., Burke A., Stickland S., McDonald J.A.K., Alexander-Hardiman K., Flanagan J., Takats Z., Cameron S.J.S. (2020). Metabolomic and metataxonomic fingerprinting of human milk suggests compositional stability over a natural term of breastfeeding to 24 months. Nutrients.

[19] García-Mantrana I., Alcántara C., Selma-Royo M., Boix-Amorós A., Dzidic M., Gimeno-Alcañiz J., Úbeda-Sansano I., Sorribes-Monrabal I., Escuriet R., Gil-Raga F. (2019). MAMI: A birth cohort focused on maternal-infant microbiota during early life. BMC Pediatrics.

[20] World Health Organization (2000). Obesity: Preventing and Managing the Global Epidemic.

[21] National Researh Council (2009). Weight Gain during Pregnancy: Reexamining the Guidelines.

[22] Boix-Amorós A., Collado M.C., Mira A. (2016). Relationship between milk microbiota, bacterial load, macronutrients, and human cells during lactation. Front. Microbiol..

[23] Cruaud P., Vigneron A., Lucchetti-Miganeh C., Ciron P.E., Godfroy A., Cambon-Bonavita M.A. (2014). Influence of DNA extraction method, 16S rRNA targeted hypervariable regions, and sample origin on microbial diversity detected by 454 pyrosequencing in marine chemosynthetic ecosystems. Appl. Environ. Microbiol..

[24] Caporaso J.G., Lauber C.L., Walters W.A., Berg-Lyons D., Lozupone C.A., Turnbaugh P.J., Fierer N., Knight R. (2010). Global patterns of 16S rRNA diversity at a depth of millions of sequences per sample. Proc. Natl. Acad. Sci. USA.

[25] García-Mantrana I., Selma-Royo M., González S., Parra-Llorca A., Martínez-Costa C., Collado M.C. (2020). Distinct maternal microbiota clusters are associated with diet during pregnancy: Impact on neonatal microbiota and infant growth during the first 18 months of life. Gut Microbes.

[26] Bolger A.M., Lohse M., Usadel B. (2014). Trimmomatic: A flexible trimmer for Illumina sequence data. Bioinformatics.

[27] Callahan B.J., McMurdie P.J., Rosen M.J., Han A.W., Johnson A.J.A., Holmes S.P. (2016). DADA2: High-resolution sample inference from Illumina amplicon data. Nat. Methods.

[28] Quast C., Pruesse E., Yilmaz P., Gerken J., Schweer T., Yarza P., Peplies J., Glöckner F.O. (2013). The SILVA ribosomal RNA gene database project: Improved data processing and web-based tools. Nucleic Acids Res..

[29] Davis N.M., Proctor D., Holmes S.P., Relman D.A., Callahan B.J. (2018). Simple statistical identification and removal of contaminant sequences in marker-gene and metagenomics data. Microbiome.

[30] Zakrzewski M., Proietti C., Ellis J.J., Hasan S., Brion M.-J., Berger B., Krause L. (2017). Calypso: A user-friendly web-server for mining and visualizing microbiome—Environment interactions. Bioinformatics.

[31] IBM Corp (2020). Released 2020. IBM SPSS Statistics for Windows, Version 27.0.

[32] Allaire J.J. (2016). RStudio: Integrated Development for R.

[33] Grote V., Verduci E., Scaglioni S., Vecchi F., Contarini G., Giovannini M., Koletzko B., Agostoni C. (2016). Breast milk composition and infant nutrient intakes during the first 12 months of life. Eur. J. Clin. Nutr..

[34] Moossavi S., Miliku K., Sepehri S., Khafipour E., Azad M.B. (2018). The prebiotic and probiotic properties of human milk: Implications for infant immune development and pediatric asthma. Front. Pediatr..

[35] Toscano M., De Grandi R., Grossi E., Drago L. (2017). Role of the human breast milk-associated microbiota on the newborns’ immune system: A mini review. Front. Microbiol..

[36] Ladomenou F., Moschandreas J., Kafatos A., Tselentis Y., Galanakis E. (2010). Protective effect of exclusive breastfeeding against infections during infancy: A prospective study. Arch. Dis. Child..

[37] Liu Y., Qin S., Song Y., Feng Y., Lv N., Xue Y., Liu F., Gosalbes M.J. (2019). The perturbation of infant gut microbiota caused by cesarean delivery is partially restored by exclusive breastfeeding. Front. Microbiol..

[38] Ho N.T., Li F., Lee-sarwar K.A., Tun H.M., Brown B.P., Pannaraj P.S., Bender J.M., Azad M.B., Thompson A.L., Weiss S.T. (2018). Meta-analysis of effects of exclusive breastfeeding on infant gut microbiota across populations. Nat. Commun..

[39] Forbes J.D., Azad M.B., Vehling L., Tun H.M., Konya T.B., Guttman D.S., Field C.J., Lefebvre D., Sears M.R., Becker A.B. (2018). Association of exposure to formula in the hospital and subsequent infant feeding practices with gut microbiota and risk of overweight in the first year of life. JAMA.

[40] Kennedy B., Peura S., Hammar U., Vicenzi S., Hedman A., Almqvist C., Andolf E., Pershagen G., Dicksved J. (2019). Oral microbiota development in early childhood. Sci. Rep..

[41] Holgerson P.L., Vestman N.R., Claesson R., Öhman C., Domellöf M., Tanner A.C.R., Hernell O., Johansson I. (2013). Oral microbial profile discriminates breastfed fron formula-fed infants. J. Pediatr. Gastroenterol. Nutr..

[42] Holgerson P.L., Esberg A., Sjödin A., West C.E., Johansson I. (2020). A longitudinal study of the development of the saliva microbiome in infants 2 days to 5 years compared to the microbiome in adolescents. Sci. Rep..

[43] Dzidic M., Collado M.C., Abrahamsson T., Artacho A., Stensson M., Jenmalm M.C., Mira A. (2018). Oral microbiome development during childhood: An ecological succession influenced by postnatal factors and associated with tooth decay. ISME J..

[44] Timby N., Domellöf M., Holgerson P.L., West C.E., Lönnerdal B., Hernell O., Johansson I. (2017). Oral microbiota in infants fed a formula supplemented with bovine milk fat globule membranes—A randomized controlled trial. PLoS ONE.

[45] Al-Shehri S.S., Sweeney E.L., Cowley D.M., Liley H.G., Ranasinghe P.D., Charles B.G., Shaw P.N., Vagenas D., Duley J.A., Knox C.L. (2016). Deep sequencing of the 16S ribosomal RNA of the neonatal oral microbiome: A comparison of breast-fed and formula-fed infants. Sci. Rep..

[46] LeMay-Nedjelski L., Asbury M.R., Butcher J., Ley S.H., Hanley A.J., Kiss A., Unger S., Copeland J.K., Wang P.W., Stintzi A. (2021). Maternal Diet and Infant Feeding Practices Are Associated with Variation in the Human Milk Microbiota at 3 Months Postpartum in a Cohort of Women with High Rates of Gestational Glucose Intolerance. J. Nutr..

[47] Solís G., de los Reyes-Gavilan C.G., Fernández N., Margolles A., Gueimonde M. (2010). Establishment and development of lactic acid bacteria and bifidobacteria microbiota in breast-milk and the infant gut. Anaerobe.

[48] Pannaraj P.S., Li F., Cerini C., Bender J.M., Yang S., Rollie A., Adisetiyo H., Zabih S., Lincez P.J., Bittinger K. (2017). Association between breast milk bacterial communities and establishment and development of the infant gut microbiome. JAMA Pediatr..

[49] Lemay-Nedjelski L., Butcher J., Ley S.H., Asbury M.R., Hanley A.J., Kiss A., Unger S., Copeland J.K., Wang P.W., Zinman B. (2020). Examining the relationship between maternal body size, gestational glucose tolerance status, mode of delivery and ethnicity on human milk microbiota at three months post-partum. BMC Microbiol..

[50] Santacruz A., Collado M.C., García-Valdés L., Segura M.T., Marítn-Lagos J.A., Anjos T., Martí-Romero M., Lopez R.M., Florido J., Campoy C. (2010). Gut microbiota composition is associated with body weight, weight gain and biochemical parameters in pregnant women. Br. J. Nutr..

[51] Heikkilä M.P., Saris P.E.J. (2003). Inhibition of *Staphylococcus aureus* by the commensal bacteria of human milk. J. Appl. Microbiol..

[52] Collado M.C., Laitinen K., Salminen S., Isolauri E. (2012). Maternal weight and excessive weight gain during pregnancy modify the immunomodulatory potential of breast milk. Pediatr. Res..

[53] Li S.W., Watanabe K., Hsu C.C., Chao S.H., Yang Z.H., Lin Y.J., Chen C.C., Cao Y.M., Huang H.C., Chang C.H. (2017). Bacterial composition and diversity in breast milk samples from mothers living in Taiwan and Mainland China. Front. Microbiol..

[54] O’Callaghan A., van Sinderen D. (2016). Bifidobacteria and their role as members of the human gut microbiota. Front. Microbiol..

[55] Selma-Royo M., Calatayud M., García-Mantrana I., Parra-Llorca A., Escuriet R., Martínez-Costa C., Collado M.C. (2020). Perinatal environment shapes microbiota colonization and infant growth: Impact on host response and intestinal function. Microbiome.

[56] Haugen M., Brantsæter A.L., Winkvist A., Lissner L., Alexander J., Oftedal B., Magnus P., Meltzer H.M. (2014). Associations of pre-pregnancy body mass index and gestational weight gain with pregnancy outcome and postpartum weight retention: A prospective observational cohort study. BMC Pregnancy Childbirth.

[57] Hunt K.M., Foster J.A., Forney L.J., Schütte U.M.E., Beck D.L., Abdo Z., Fox L.K., Williams J.E., McGuire M.K., McGuire M.A. (2011). Characterization of the diversity and temporal stability of bacterial communities in human milk. PLoS ONE.

[58] Li R., Jewell S., Grummer-Strawn L. (2003). Maternal obesity and breast-feeding practices 1,2. Am. J. Clin. Nutr..

[59] Ojo-Okunola A., Claassen-Weitz S., Mwaikono K.S., Gardner-Lubbe S., Stein D.J., Zar H.J., Nicol M.P., Du Toit E. (2019). Influence of socio-economic and psychosocial profiles on the human breast milk bacteriome of south african women. Nutrients.

[60] Padilha M., Brejnrod A., Danneskiold-Samsøe N.B., Hoffmann C., Iaucci J.d.M., Cabral V.P., Xavier-Santos D., Taddei C.R., Kristiansen K., Saad S.M.I. (2020). Response of the human milk microbiota to a maternal prebiotic intervention is individual and influenced by maternal age. Nutrients.

