# Supplementary material for: Breastfeeding Practices Influence the Breast Milk Microbiota Depending on Pre-Gestational Maternal BMI and Weight Gain over Pregnancy
Source: Nutrients. 2021 Apr 30;13(5):1518. doi: 10.3390/nu13051518 (PMC8146841; doi:10.3390/nu13051518)
Supplement: Supplementary file 1 [file nutrients-13-01518-s001.zip › nutrients-1188754-supplementary.pdf]

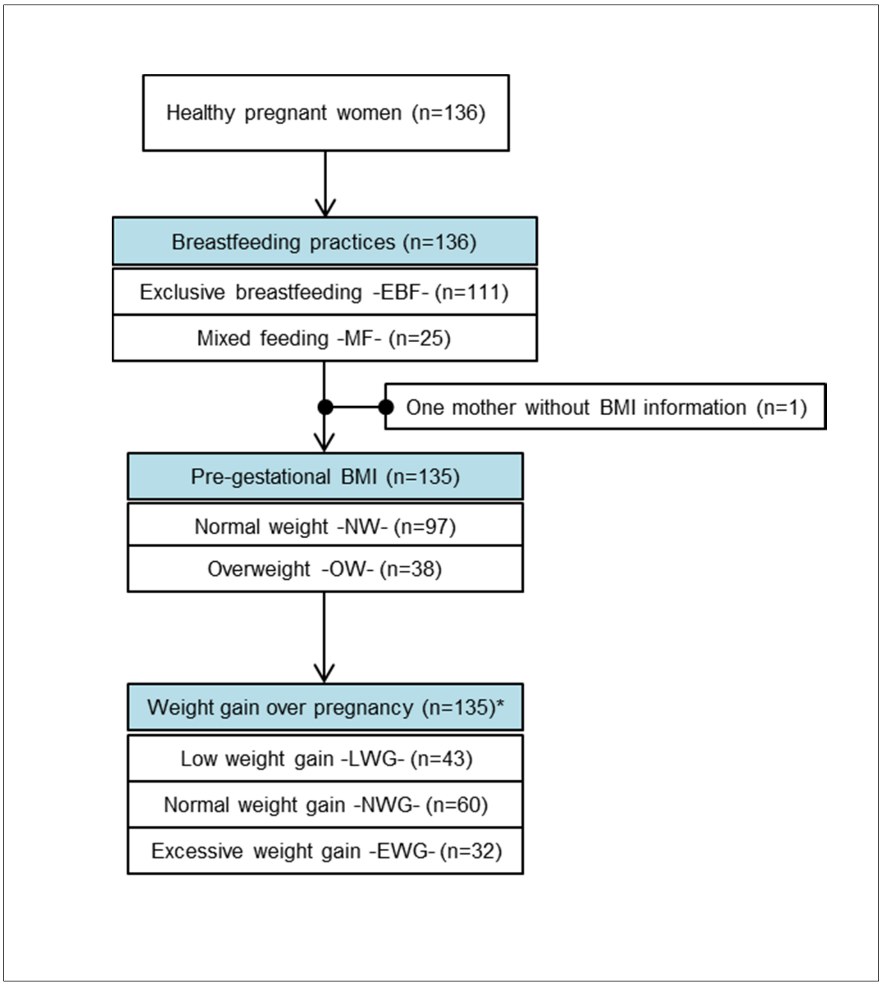

Figure S1. Flow diagram of study participants. *Low weight gain (LWG) data were not used in the analysis.

Table S1. Poisson regression model for top 10 genera relative abundance based on pre-gestational BMI and weight gain over pregnancy.

		Pre-gestational BMI		
**Genus**		**IRR**	**95 % CI**	***p***
Streptococcus	NW vs OW	1.21	1.12-1.30	**<0.001**
Staphylococcus	NW vs OW	0.89	0.83-0.96	**0.002**
Ralstonia	NW vs OW	1.16	1.03-1.32	**0.017**
Acinetobacter	NW vs OW	0.94	0.83-1.06	0.326
Gemella	NW vs OW	0.97	0.81-1.16	0.718
Pseudomonas	NW vs OW	0.55	0.44-0.68	**<0.001**
Lactobacillus	NW vs OW	0.94	0.75-1.19	0.611
Escherichia/Shigella	NW vs OW	1.50	1.02-2.17	**0.037**
Bifidobacterium	NW vs OW	4.67	2.53-8.64	**<0.001**
Stenotrophomonas	NW vs OW	12.0	4.90-29.20	**<0.001**
		**Weight gain over pregnancy**		
Streptococcus	NWG vs EWG	1.38	1.27-1.51	**<0.001**
Staphylococcus	NWG vs EWG	1.00	0.92-1.09	0.942
Ralstonia	NWG vs EWG	0.53	0.46-0.61	**<0.001**
Acinetobacter	NWG vs EWG	0.80	0.68-0.93	**0.004**
Gemella	NWG vs EWG	1.36	1.09-1.70	**0.006**
Pseudomonas	NWG vs EWG	8.14	5.33-12.42	**<0.001**
Lactobacillus	NWG vs EWG	0.60	0.46-0.79	**<0.001**
Escherichia/Shigella	NWG vs EWG	0.19	0.14-0.26	**<0.001**
Bifidobacterium	NWG vs EWG	3.20	1.71-5.98	**<0.001**
Stenotrophomonas	NWG vs EWG	0.24	0.11-0.54	**<0.001**

Poisson regression models were run for top 10 genera based on pre-gestational BMI and weight gain, adjusted for mode of birth and breastfeeding practices at one month. $p < 0.05$ was considered statistically significant. IRR. Incidence rate ratio. CI. Confidence interval. (NW) Normal weight and (OW) Overweight. (EWG) Excessive weight gain and (NWG) Normal weight gain.

Table S2. Characteristics of population participant based pre-gestational BMI (n=135)

	All	NW (18.5 – 24.9 kg/m²) n=97	OW (> 25 kg/m²) n=38	*p*
**Maternal characteristics**				
Maternal age (years)	34.44 ± 3.80	34.25 ± 3.82	34.95 ± 3.74	0.338
Gestational age (weeks)	40 [39-40]	40 [39-40]	40 [39-40]	0.604
Pre-gestational BMI (kg/m²)	22.84 [21.01-25.39]	21.63 [20.46-22.96]	27.25 [25.97-29.69]	**<0.001**
Weight gain (kg) over pregnancy*	12 [9.5-15]	12 [10-15]	10 [7.8-14]	**0.023**
Low weight gain (LWG)	43 (31.9)	36 (37.1)	7 (18.4)	**0.004**
Normal weight gain (NWG)	60 (44.4)	45 (46.4)	15 (39.5)	
Excessive weight gain (EWG)	32 (23.7)	16 (16.5)	16 (42.1)	
Intrapartum antibiotic exposure (%)	54 (40)	35 (36.1)	19 (50)	0.138
Antibiotic during pregnancy (%)	42 (31.1)	27 (27.8)	15 (39.5)	0.189
Antibiotic treatment 1 month (%)	11 (8.2)	8 (8.3)	3 (7.9)	0.934
**Infant characteristics**				
Gender Female (%)	76 (56.3)	55 (56.7)	21 (55.3)	0.965
Birth mode: vaginal birth (%)	84 (62.2)	65 (67)	19 (50)	**0.046**
Height at birth (cm)	49.86 ± 2.10	49.75 ±2.16	50.16 ±1.95	**0.309**
Weight at birth (g)	3.32 ± 0.44	3.26 ±0.43	3.48 ± 0.45	**0.009**
Antibiotic treatment 1 month (%)	8 (5.9)	5 (5.2)	3 (7.9)	0.544
Breastfeeding duration (months)	8.37 ± 3.92	8.43 ± 3.89	8.20± 4.04	0.755
Exclusive Breastfeeding (1 month)	110 (81.5)	79 (81.4)	31 (81.6)	0.985

Categorical data results are shown as number of case (percentage %). Normally distributed data are shown as mean ± SD and non-normal data as median [interquartile range= IQR]. (NW) Normal weight, (OW) Overweight. (NWG) Normal weight gain, (EWG) Excessive weight gain.* Low weight gain (LWG) data were not used in the analysis.

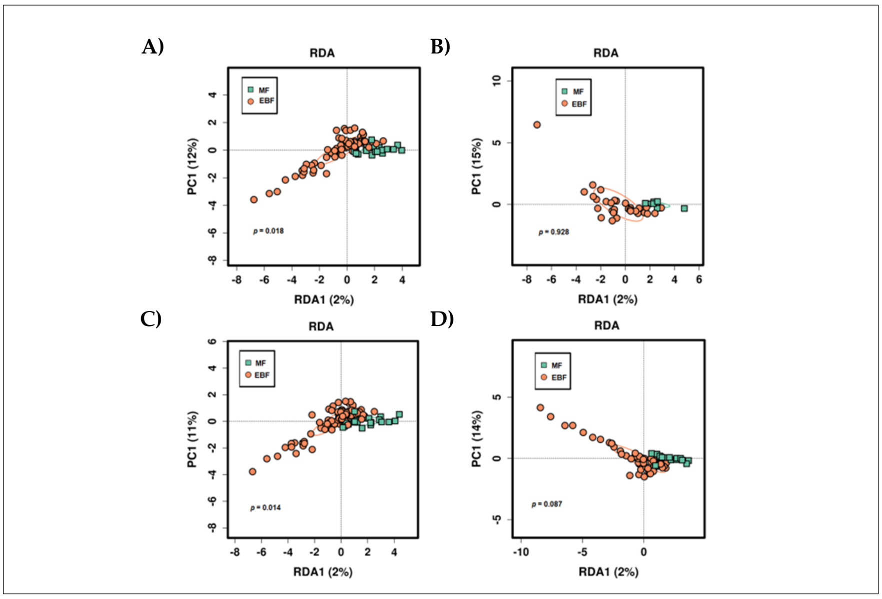

Figure S2. Impact of pre-gestational BMI and weight gain over pregnancy on breast milk microbiota according to breastfeeding practices. Multivariate redundancy discriminant analysis (RDA) showed significant differences in breast milk microbial communities between breastfeeding practices depending on pre-gestational BMI: (A) NW mothers (B) and OW mothers. Weight gain over pregnancy: (C) NWG mothers and (D) EWG mothers. (EBF) Exclusive breastfeeding and (MF) Mixed feeding.

Table S3. Comparison of breast milk microbiota composition at top 10 genera, by breastfeeding practices and pre-gestational BMI and Weight gain.

Genus	EBF_NW	EBF_OW	MF_NW	MF_OW	*p*
Streptococcus	28.99 [8.49-47.91]	16.69 [6.42-35.92]	27.84 [10.92-48.37]	26.35 [7.02-61.14]	0.363
Staphylococcus	19.69 [5.25-38.86]	23.07 [4.47-40.50]	26.91 [13.12-57.48]	56.36 [8.09-67.54]	0.323
Ralstonia	1.14 [0-21.62]	0.095 [0.01-3.59]	0.075 [0-2.11]	0.01 [0-4.82]	0.216
Acinetobacter	0.39 [0.07-1.54]	0.20 [0.02-26.59]	0.18 [0-6.82]	0.11 [0.02-1.81]	0.502
Gemella	1.39 [0.16-4.42]	1.00 [0.29-6.21]	1.86 [0.46-5.90]	4.58 [0.92-5.17]	0.804
Pseudomonas	0.57 [0.21-1.89]	**0.63 [0.11-2.13]**	0.12 [0.03-0.48]	0.09 [0.04-0.25]	**<0.01**
Lactobacillus	0.51 [0.03-4.18]	0.92 [0.07-2.80]	0.48 [0-1.30]	0.2[0-1.11]	0.319
Escherichia/Shigella	0.05 [0-0.17]	0.04 [0-0.25]	0.05 [0-0.18]	0 [0-0.14]	0.738
Bifidobacterium	**0.30 [0.05-1.10]**	0.075 [0.03-0.58]	0.07 [0.01-0.20]	0.05 [0-0.63]	**0.033**
Stenotrophomonas	0.08 [0-0.32]	0.045 [0-0.29]	0.04 [0-0.08]	0.01 [0-0.04]	0.186
Genus	EBF_NWG	EBF_EWG	MF_NWG	MF_EWG	*p*
Streptococcus	31.04 [11.23-51.44]	17.67 [6.81-29.73]	27.83 [12.93-44.33]	26.35 [6.50-54.40]	0.298
Staphylococcus	21.98 [6.55-41.65]	21.44 [4.04-31.49]	**44.01 [24.02-67.54]**	26.39 [16.05-78.23]	**0.049**
Ralstonia	0.44 [0-9.05]	0.10 [0.02-29.48]	0.01 [0-0.08]	0.03[0-10.29]	0.225
Acinetobacter	0.13 [0.03-1.16]	0.34 [0.06-7.98]	0.11 [0.01-4.35]	0.02 [0-0.16]	0.076
Gemella	3.08 [0.17-6.78]	1.08 [0.29-4.42]	4.72 [0.20-6.91]	5.35 [2.68-13.29]	0.402
Pseudomonas	0.50 [0.14-1.76]	**0.86 [0.15-1.65]**	0.09 [0.02-0.37]	0.10 [0.04-0.30]	**0.019**
Lactobacillus	0.82 [0.03-2.59]	1.09 [0.04-7.88]	0.58 [0.02-1.07]	0.39 [0-1.23]	0.438
Escherichia/Shigella	0.06 [0-0.19]	0.07 [0.01-0.18]	0.08 [0-0.18]	0.02 [0.01-28.78]	0.983
Bifidobacterium	0.15 [0.03-1.10]	0.24 [0.04-1.04]	0.06 [0.02-0.27]	0.08 [0.03-0.19]	0.436
Stenotrophomonas	0.05 [0-0.16]	0.08 [0-0.59]	0.03 [0-0.07]	0 [0-0.03]	0.063

Relative abundances of dominant genus within each breastfeeding practices and pre-gestational BMI and weight gain over pregnancy are in bold. Data is presented as median [interquartile range= IQR]. Relative abundances were compared by Mann-Whitney test.

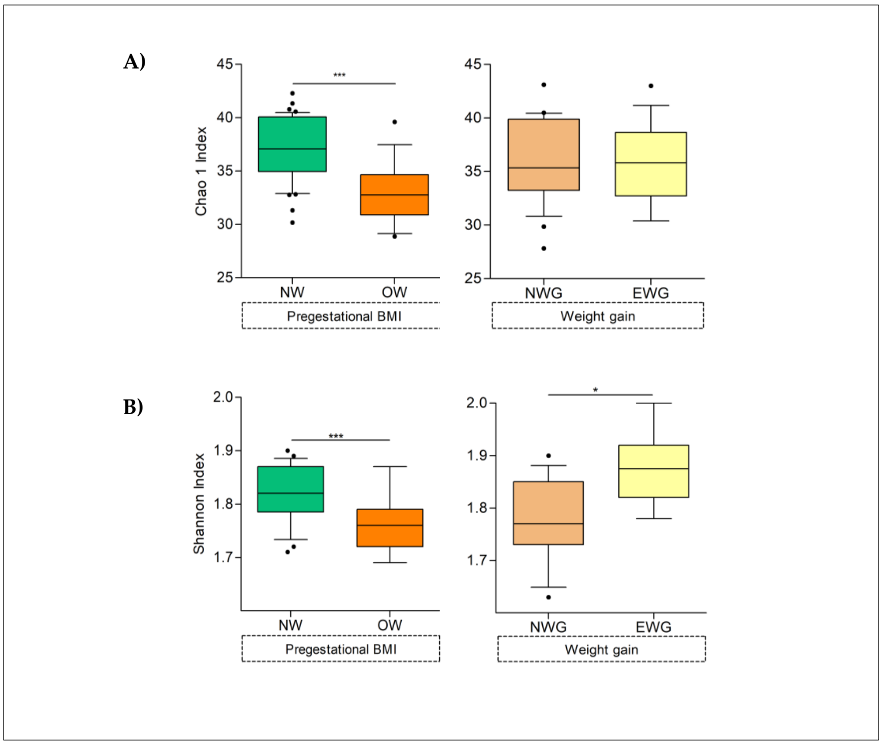

Figure S3. Microbial richness and diversity is associated by pre-gestational BMI and weight gain influence. Diversity (Shannon index) and richness (Chao1 index) of breast milk microbiota according to pre-gestational BMI and weight gain categories. Microbial richness and diversity indexes at ASV. (A) Richness and according to pre-gestational BMI and weight gain. (B) Diversity according pre gestational BMI and weight gain. Richness and diversity values were adjusted for total bacterial load. Whiskers represented 5-95 percentile interval. * p <0.05, **p <0.01, ***p <0.001. (NW) Normal weight, (OW) Overweight. (EWG) Excessive weight gain and (NWG) Normal weight gain

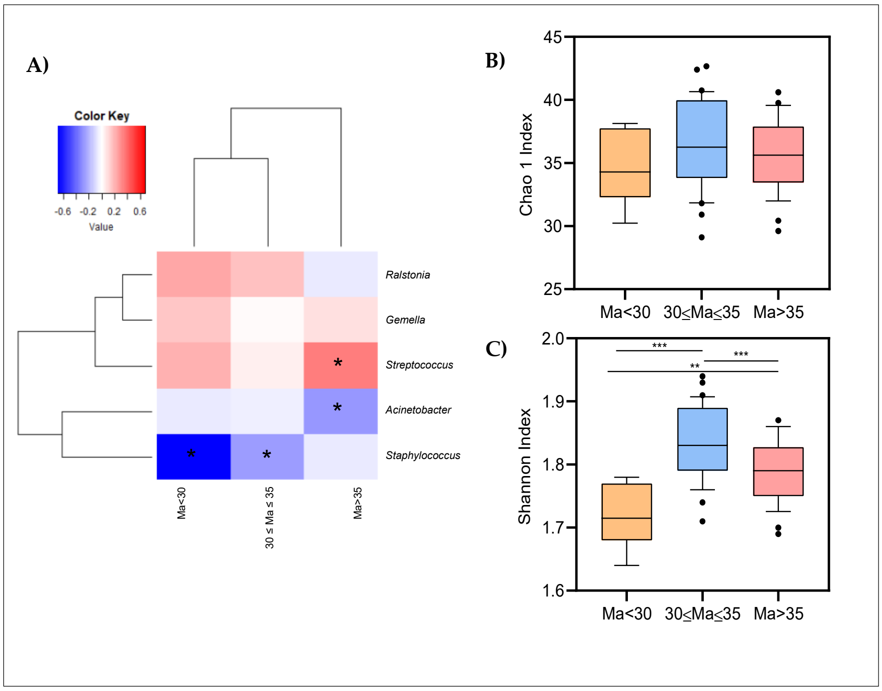

Figure S4. Impact of maternal age on breast milk microbiota. (A) Maternal age was associated with the relative abundances of top 5 genera (Spearman's rank correlation). (B, C) Microbial richness and diversity indexes at ASV. Richness and diversity values were adjusted for total bacterial load. Whiskers represented 5-95 percentile interval. * *p* <0.05, ***p* <0.01, ****p* <0.001.
